# Long‐term oral administration of burdock fructooligosaccharide alleviates DSS‐induced colitis in mice by mediating anti‐inflammatory effects and protection of intestinal barrier function

**DOI:** 10.1002/iid3.1092

**Published:** 2023-11-22

**Authors:** Qunfei Ma, Xiujuan Zhang, Xuan Xu, Yan Lu, Qiang Chen, Yiru Chen, Chunyan Liu, Kaoshan Chen

**Affiliations:** ^1^ School of Life Science Shandong University Qingdao China; ^2^ Department of Physiology Naval Medical University Shanghai China; ^3^ Clinical Laboratory Medicine Department Jining No. 1 People's Hospital Jining China; ^4^ Burdock Biotechnology (Dezhou) Co., Ltd Dezhou China; ^5^ Provincial Engineering Laboratory for Screening and Re‐Evaluation of Active Compounds of Herbal Medicines in Southern Anhui, School of Pharmacy Drug Research & Development Center, Anhui Provincial Engineering Research Center for Polysaccharide Drugs, Wannan Medical College Wuhu China

**Keywords:** burdock fructooligosaccharide, colitis, DSS, inflammatory cytokines, intestinal barrier

## Abstract

**Background:**

Ulcerative colitis, a typical subtype of inflammatory bowel disease, can cause many serious complications. Burdock fructooligosaccharide (BFO), a linear inulin with a purity of 99.439% and a molecular weight of 2345 Da, demonstrates anti‐inflammatory and immunomodulatory properties.

**Methods:**

The Kunming mice were divided into two experimental models: a normal pretreatment model and a colitis experimental model. During the experimental treatment period, we assessed changes in weight and disease activity index (DAI), quantified the intestinal index, and determined myeloperoxidase (MPO) activity and reactive oxide species (ROS) levels in colitis mice. We also photographed colon morphology to investigate alterations in the integrity of the intestinal barrier function. Finally, we performed ELISA and qRT‐PCR to evaluate the anti‐inflammatory effect of BFO treatment on colitis mice.

**Result:**

The long‐term oral administration of BFO alone exhibited protective effects by preventing disruption of the intestinal functional structure and increasing the colon index in mice. However, in a dextran sodium sulfate (DSS)‐induced colitis mouse model, BFO administration facilitated quick recovery of body weight and effectively reduced the DAI, especially in the BFO‐H group (500 mg/kg/day). BFO treatment maintained the integrity of the intestinal barrier by attenuating the crypt distortion and increasing the goblet cells count It restored the DSS‐induced colon shortening and reduced the symptoms of colitis. These effects may be attributed to the appropriate concentrations of BFO effectively inhibiting MPO activity, clearing excessive ROS, and relieving spleen abnormalitie. BFO also attenuated the overexpression and excessive secretion of inflammatory cytokines (TNF‐α, IL‐1β, IL‐6, and MCP‐1) induced by DSS, reduced intestinal inflammation, and consequently protected the intestinal barrier function.

**Conclusion:**

BFO effectively alleviated the symptoms of DSS‐induced colitis by mediating anti‐inflammatory effects and protecting the intestinal barrier integrity, thereby potentially facilitating the utilization of safer and more efficacious polysaccharides for managing chronic inflammatory diseases.

## Graphical abstract

Burdock fructooligosaccharide (BFO) attenuated the overexpression of inflammatory cytokines induced by dextran sodium sulfate (DSS). BFO treatment prevented the disruption of the intestinal functional structure. BFO can effectively reduce the severity of DSS‐induced colitis.

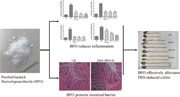

## 1 INTRODUCTION

Ulcerative colitis (UC), a typical inflammatory bowel disease (IBD), is a chronic remittent or progressive intestinal inflammatory disease with a high relapse rate.^1^, ^2^ While the etiology of UC is complex, its pathogenesis involves genetic susceptibility, irregular diet and rest, abnormal autoimmunity, environmental pollutants, and antibiotic abuse.^3^, ^4^ UC is often associated with weight loss, diarrhea, abdominal pain, and bloody stool, owing to the damage caused to the intestinal mucosal structure, and is pathologically characterized by a decrease in the number of goblet cells, an increase in inflammatory cell infiltration, crypt abscess,^5^ and high risk of colitis‐associated cancer.^6^ The damage caused to the intestinal barrier may increase the intestinal permeability, thereby leading to bacterial invasion and tissue inflammation.^7^ Furthermore, the excessive secretion of inflammatory cytokines aggravate the damage caused to the intestinal barrier function and is closely related to the occurrence and development of IBD.^8^

The incidence of UC is gradually increasing worldwide and tending to rise among the younger population, thus seriously affecting their daily lives and physical and mental health.^9^ While there is a surge in the availability of anti‐colitis drugs such as corticosteroids, immunosuppressants, and anti‐inflammatory and bactericidal agents, their excessive and long‐term utilization may lead to serious side‐effects, severe gastrointestinal reactions, and even symptoms of multiple drug intolerance.^9^, ^10^ Therefore, a novel drug with high efficiency and low toxicity is warranted for prevention and treatment of colitis, one of the major public health issues that need urgent attention.

The improvement in the quality of life has encouraged people to pay more attention to their intestinal health. Numerous studies have highlighted the role of biomolecules (such as polyphenols, glycosides, and polysaccharides) from natural extracts in protection of colitis in mice.^11^, ^12^, ^13^ Among these natural extracts, polysaccharide are macromolecular carbohydrates composed of aldose or ketose molecules linked by a glycosidic bond that exhibit pharmacological activities such as anti‐inflammatory, antitumor, antidiabetic, and immunomodulatory properties.^14^, ^15^ Polysaccharides offer the advantages of high safety, good efficacy, low cost, and minor adverse reactions, and have been proven to exert protective effects on intestinal health.^16^, ^17^

Burdock (*Arctium lappa* L.) belongs to the Compositae family and is consumed as a food and medicinal product,^18^ for the prevention of metabolic diseases, boosting immunity, and effectively invigorating health.^19^ de Almeida et al.^20^ reported that the lactone sesquiterpene onopordopicrin‐enriched fraction (ONP fraction), extracted from the leaves of *A. lappa*, showed anti‐inflammatory activity in the intestine of a TNBS colitis model. Ji et al.^21^ demonstrated that *A. lappa* extracts improved DSS‐induced IBD by enhancing immune responses. However, these extracts were complex mixtures derived from either burdock leaves or roots, thus containing multiple substances. In contrast, BFO utilized in the present study was extracted and purified from burdock root. It belongs to the GFn inulin‐type with a linear chain structure, consisting of d‐fructose and d‐glucose at a molar ratio of 12:1. The linkage among d‐fructose is established by a β (2 → 1) glucosidic bond, while d‐glucose is connected though an α (1 → 2) glucosidic bond at the end of the backbone. The average molecular weight is approximately 2357 Da.^22^ Although a water‐soluble GFn inulin‐type polysaccharide (ALP‐1) purified from *A. lappa* was found by Wang et al.^23^ to alleviate mouse colitis, this polysaccharide had a molecular weight of 5120 Da and possessed branched chains, differing from the polysaccharide used in the present study, which may be related to different extraction methods.^24^ Our previous research had found that BFO effectively improved the immunological function of S180 tumor‐bearing mice.^18^ Herein, we aimed to investigate the preventive effects of BFO in colitis and elucidate the underlying mechanism.

We preliminarily explored the effect of BFO on the intestinal barrier function and alleviation of dextran sodium sulfate (DSS)‐induced colitis in mice. DSS, a polysaccharide sulfate widely used to establish UC models, induced clinical symptoms and histological characteristics similar to those observed in human UC.^25^, ^26^ We evaluated the protective effects of BFO on DSS‐induced colitis in mice by analyzing the disease activity index (DAI) score, performing histological analysis, and studying the anti‐inflammatory mechanism. Our results may provide a theoretical basis for future studies elucidating the molecular mechanism of action of natural polysaccharides in preventing UC, and promote development and application of BFO‐based therapeutic agents for colitis treatment.

## 2 MATERIALS AND METHODS

### 2.1 Materials and reagents

Fresh burdock Root was purchased from Jimo vegetable farm center and identified at School of Life Science, Shandong University. Dextran Sulfate Sodium Salt (DSS) with a molecular weight of approximately 40,000 were purchased from Yuanye Biotechnology Co. Ltd. TRIzol Reagent was purchased from Beyotime Biotechnology. Experimental mice Kunming were provided by Qingdao Daren Fucheng Animal Technology Co. Ltd.

### 2.2 Preparation and purification of BFO

The methods for the preparation and purification of BFO were referenced from Hao et al.^22^ and suitably modified accordingly. Briefly, fresh burdock roots were cut into pieces after thorough cleaning and peeling, followed by immersion in hot water at a temperature of 70°C with a ratio of 1:10 (m/v) for 90 min. After filtration using a Buchner funnel, the filtrate was subjected to decolorization with D301R macroporous resin and subsequently concentrated to one‐fifth of its original volume. The resulting concentrated solution was then mixed with four times its volume of 95% ethyl alcohol and stay in 4°C at least 12 h. The precipitate was collected and subjected to sequential washing with ether, acetone, and anhydrous ethanol. Subsequently, the organic solvent was removed and the precipitate was fully redissolved using deionized water. Insoluble impurities were separated by centrifugation, followed by deproteinization of the supernatant using the Sevage method. The residual organic solvent was evaporated, and the resulting solution was concentrated and freeze‐dried to obtain crude BFO.

The crude BFO was initially purified using gel filtration chromatography on a Sephadex G50 (Solarbio) column (1.5 cm × 60 cm) with distilled water flowing at a rate of 0.5 mL/min. The polysaccharide peak from the chromatogram was collected and freeze‐dried for further purification using a Sephacryl S‐200 h column (1.6 × 60 cm) with distilled water flowing at a rate of 0.35 mL/min. Subsequently, the isolated polysaccharide peak was collected and freeze‐dried for purity assessment and activity determination.

The purity assay was conducted using a Shimadzu HPLC system equipped with a refractive index detector (RID) and G3000PWXL chromatographic columns (7.8 × 300 mm, TSK gel). The analysis was performed with ultra‐pure water at a flow rate of 1.0 mL/min. A sample volume was 10 μL with a concentration of 10 mg/mL was injected, and the temperature was 40°C.

### 2.3 Experimental design for the mice normal and colitis model

Kunming mice (female, 6 weeks old) were procured from Daren Fucheng Animal Husbandry Co. Ltd, renowned for their robust disease resistance and adaptability, making them extensively employed in various research disciplines such as pharmacology and toxicology. All animals were housed in a pathogen‐free environment with controlled temperature (25 ± 1°C), humidity (55 ± 5%), and photoperiod (12 h light/12 h dark). After acclimatization for 1 week, the mice were divided into two experimental models:

Normal experimental model ‐ randomly assigned to four experimental groups (*n* = 10/group) without DSS administration. The CK (control group), pre‐BFO‐L (low concentration pretreatment), pre‐BFO‐M (medium concentration pretreatment), and pre‐BFO‐H (high concentration pretreatment) groups received deionized water and BFO solution at doses of 100, 250, and 500 mg/kg/day respectively. The mice were fed a standard diet throughout the study period. All mice had ad libitum access to drinking water for a duration of 56 days.

**Figure 1:**
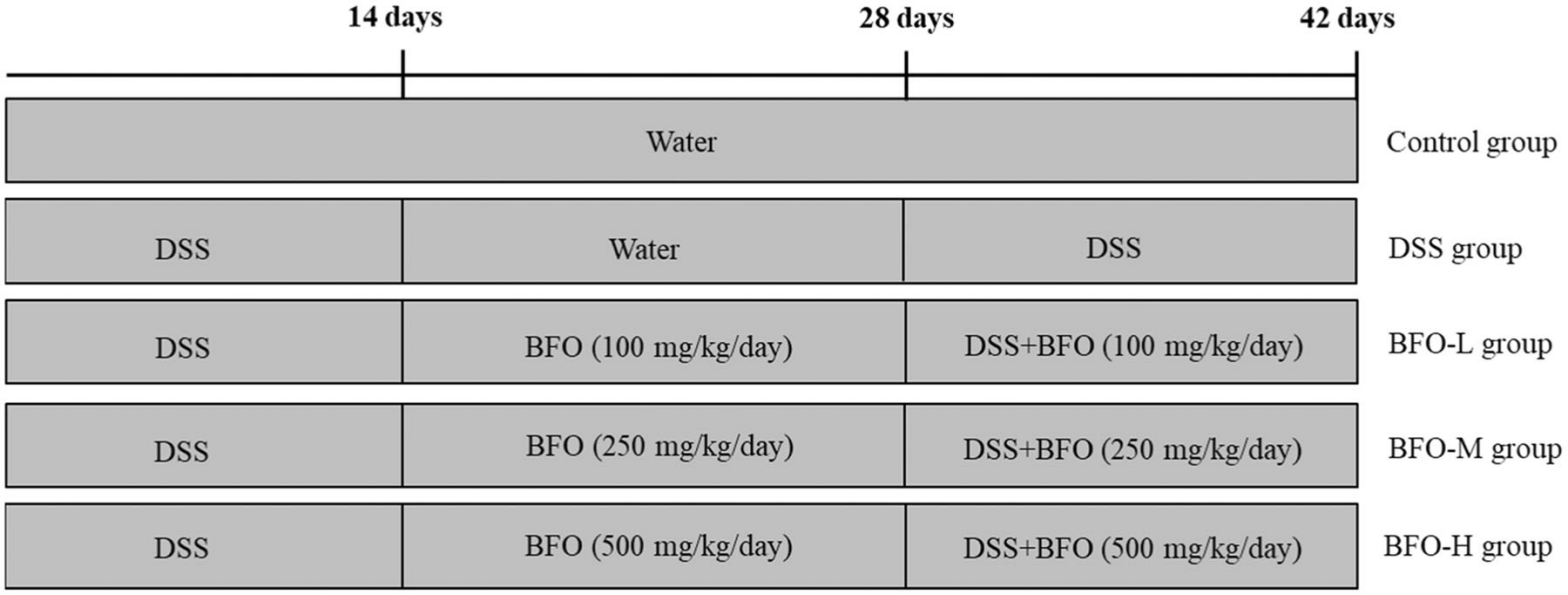
The experimental design of the dextran sodium sulfate (DSS)‐induced colitis in mice.

Colitis experimental model—following the protocol described by Wang et al.^27^ with appropriate modifications; the mice were randomly allocated into five experimental groups (*n* = 10/group) as illustrated in Figure 1. The experiment was divided into the control group (CK), DSS group, BFO‐L treatment group (100 mg/kg/day of BFO), BFO‐M treatment group (250 mg/kg/day of BFO), and BFO‐H treatment group (500 mg/kg/day of BFO). Throughout the experimental period, the CK group was provided with deionized water as their diet (*n* = 10/group). In the first stage of the experiment, the DSS group (*n* = 40/group) were fed with DSS water (3% w/v) for 14 days. Subsequently, in the second stage, DSS water was fasted for 14 days to allow for recovery. During this period, mice in the DSS group of the first stage were randomly assigned to four groups (DSS, BFO‐L, BFO‐M, and BFO‐H; *n* = 10/group), and correspondingly administered deionized water or 100, 250, and 500 mg/kg/day of BFO, respectively. In the third stage, treatment groups (DSS, BFO‐L, BFO‐M, and BFO‐H groups) were readministered 3% DSS water for 14 days, while concurrently supplementing the BFO treatment groups with 100, 250, and 500 mg/kg/day of BFO, respectively. The mice were fed a standard diet. All mice were allowed to freely drink the respective solutions for 42 days. The selection of BFO concentration was based on our preliminary experiment and previous research.^18^, ^28^

### 2.4 Assessment of disease activity index (DAI)

DAI was determined following the method of Liu et al.^29^ and appropriately modified. Weight change, fecal occult blood, and stool trait were recorded to calculate the DAI score according to Table 1 for assessing the disease severity (*n* = 10/group). Fecal blood levels were measured using a fecal occult blood qualitative test kit (pyramidon method) (Yuanye Biotechnology Co. Ltd.) according to the instructions. DAI = (weight change score + fecal blood score + stool trait score)/3.

**Table 1:** The score of disease activity index.

Score	Weight change (%)	Fecal occult blood	Stool trait
0	None	No bleeding	Normal
1	1–5	Occult blood (negative)	Loose stools (forming)
2	5–10	Occult blood (positive)	Loose stools (no forming)
3	10–15	Bleeding	Diarrhea (slight)
4	*>*15	Gross bleeding	Diarrhea (serious)

### 2.5 Determination of intestinal index

At the end of the experiment, the mice were euthanized. Small intestine, cecum, and colorectal were separated and washed with PBS before being drained using a sterile filter paper. Subsequently, the colon in the experimental model of colitis was photographed and measured using a ruler. Furthermore, the weights of the small intestine cecum, colorectal tissue, and entire intestinal tract were measured. The colon in colitis experimental model was photographed and measured using a ruler. The indices of the small intestine, cecum, colon, and whole intestine were calculated as follows: the index of the small intestine or whole intestine = the weight of the small intestine or whole intestine/the weight of the mouse × 100%. The measurements were repeated seven times in each group.

### 2.6 Analysis of the colon morphology of colitis mice

The colon tissues from each mouse in the normal and colitis experimental models were sectioned into 5 mm pieces and fixed overnight in a 4% paraformaldehyde solution. Subsequently, the tissue was embedded in paraffin, cut into 5 μm thick sections, and stained with hematoxylin/eosin (H&E, Beyotime Biotechnology) for microscopic observation and photography. Each group was evaluated five times.

### 2.7 Determination of myeloperoxidase (MPO) activity of colitis mice

Approximately 20 mg of colon tissues were homogenized in an ice bath to determine the MPO activity according to the instructions of the myeloperoxidase assay kit (Nanjing Jiancheng). The absorbance was measured at 460 nm by a microplate reader (Epoch 2, Bio Tek Instruments). The measurements were repeated three times in each group.

### 2.8 Determination of reactive oxide species (ROS) of colitis mice

ROS in colon tissues was determined using ROS Assay Kit (Nanjing Jiancheng) according to the manufacturer's instructions. Briefly, the cell suspensions obtained from digested colon tissue were collected and incubated with 2′,7′‐dichlorofluorescein diacetate (DCF‐DA) at 37°C for 1 h. Fluorescence intensity analysis was performed using a microplate spectrometer (ENSPIE, PE). The measurements were repeated three times in each group.

### 2.9 Quantitative real‐time polymerase chain reaction (qRT‐PCR)

The experiment was performed by referring to the method described by Song et al.^30^ Briefly, colon tissues were ground into powder using liquid nitrogen by a mortar, followed by isolation of total RNA using TRIzol reagent (Beyotime Biotechnology). Subsequently, cDNA synthesis was performed from the isolated total RNA using a 5× All‐In‐One RT Master Mix kit (Abm Inc.). The primer sequences are listed in Table 2. The qRT‐PCR was performed on the LightCycler® 480 Instrument following the manufacturer's instructions for the SYBR Green Master Mix kit (DBI Bioscience). The relative expression level of the target genes was calculated using the 2^−ΔΔCt^ calculation method. Values were normalized to the internal control NADPH. Each group was evaluated three times.

**Table 2:** A sequence of genetic primers applicable to the mice aorta and liver.

Gene	Forward	Reverse
IL‐1β	TGAAATGCCACCTTTTGACAGTGAT	GCCTGCCTGAAGCTCTTGTTG
COX‐2	CAACACCTGAGCGGTTACCA	AGAGGCAATGCGGTTCTGAT
INOS	CAGCTGGGCTG ACAAACCTT	CATTGGAAGTGAAGCGTTTCG
TNF‐α	GAACTCCAGGCGGTGCCTAT	GGTGGTTTGTGAGTGTGAGGGT
IL‐6	ATGGATGCTACCAAACTGGAT	TGAAGGACTCTGGCTTTGTCT
NADPH	AGTATGTCGTGGAGTCTA	AATCTTGAGTGAGTTGTC

### 2.10 Measurement of inflammatory response of colitis mice

At the end of the experiment, the contents of the tumor necrosis factor‐α (TNF‐α), interleukin (IL)−6, and IL‐10 in serum were determined following the manufacturer's instructions provided with the mouse ELISA kits (Elabscience Biotechnology Co. Ltd.). Protein concentration was measured with a BCA assay kit (Beyotime Biotechnology Industry).

The spleens of the experimental mice were collected and weighed to calculate the spleen index according to the following formula: the spleen index = the weight of the spleen (g)/the weight of the mouse (g) × 100%.

### 2.11 Statistical analyses

The statistical analysis was performed using GraphPad Prism 8.4 software. Experimental results were presented as the mean ± standard deviation. *T*‐test was employed to determine significant differences between two samples, while one‐way analysis of variance (ANOVA) with Tukey's HSD test was used for multiple comparison tests. Significant differences between groups were denoted as *p < .05, **p < .01, ***p < .001 versus CK group; and ^#^ *p* < .05, ^##^ *p* < .01, ^###^ *p* < .001 versus DSS group based on the findings obtained from at least three independent measurements for each group.

## 3 RESULTS

### 3.1 Assessment of BFO purity

**Figure 2:**
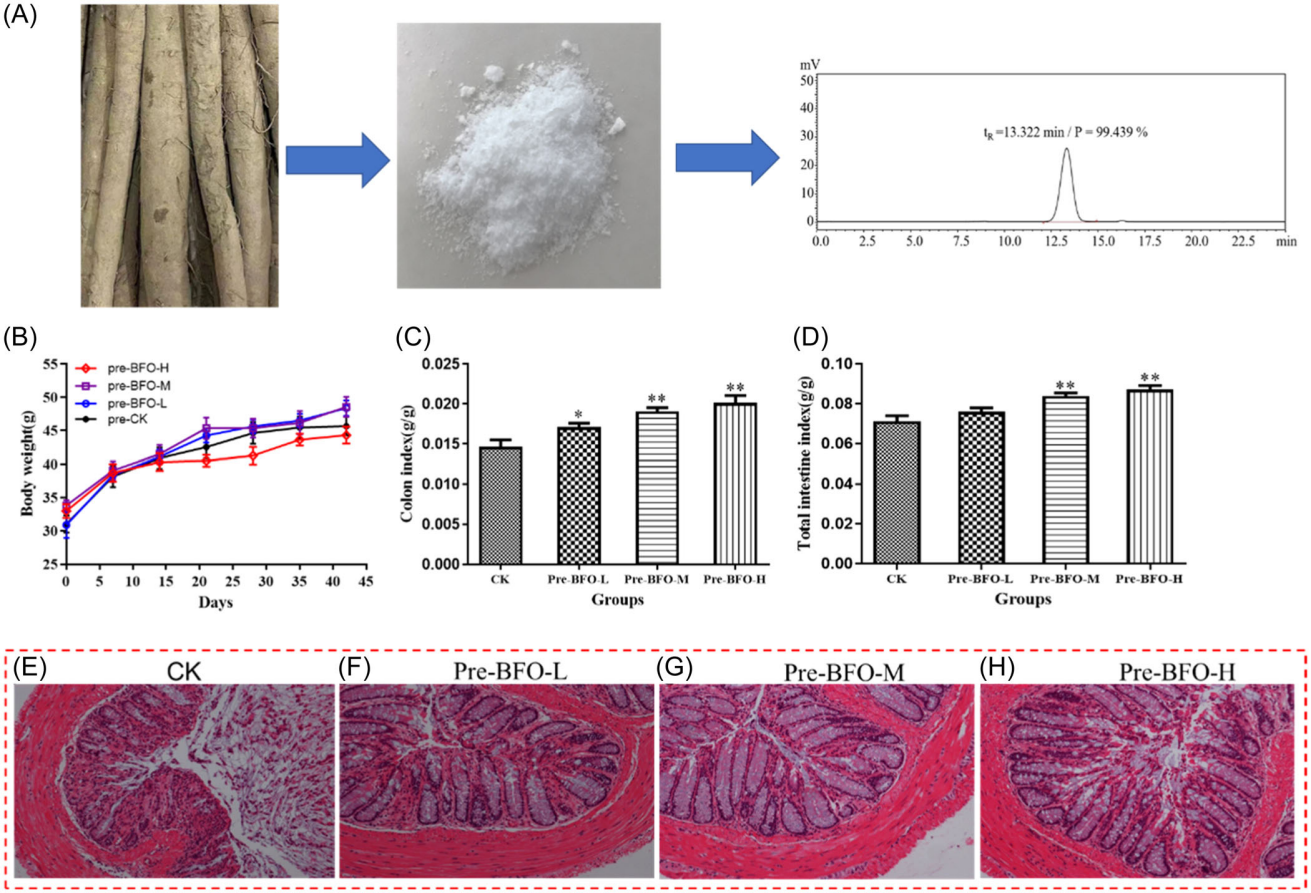
Effects of burdock fructooligosaccharide (BFO) on the colon of a normal mice model. (A) Extraction and liquid chromatographic purity of BFO extracted from burdock root; (B) body weight changes; (C) colon index; (D) total intestinal index; and the morphology of colon tissues with H&E staining in the CK group (E), pre‐BFO‐L group (100 mg/kg/day) (F), pre‐BFO‐M group (250 mg/kg/day) (G), and pre‐BFO‐H group (500 mg/kg/day) (H). Experiments were repeated at least 10 times, data are presented as the mean ± standard deviation (SD). *p < .05, **p < .01 versus CK group (analysis of variance).

The BFO eluted from a gel filtration column was collected as a single peak, concentrated, and freeze‐dried. The resulting product was BFO in the form of a white powder (Figure 2A). The purity of BFO was determined by high‐performance liquid chromatography (HPLC), wherein a single symmetric peak of BFO was obtained at a retention time of 13.322 min and a purity value of 99.439%.

### 3.2 Effect of BFO treatment on normal mice

Before the administration of DSS, we studied the effects of BFO on the body weight, colon index, and whole intestine index of normal mice. Long‐term administration of different concentrations of BFO (100, 250, and 500 mg/kg/day) had no significant effect on the body weight of mice (vs. the CK group; Figure 2B). Although the body weight of the mice from the pre‐BFO‐H group was lower than that of the mice from the CK group from day 20 onward, the difference was not significant (*p* > .05).

However, the long‐term administration of different concentrations of BFO resulted in an elevation of the colon index (Figure 2C) and whole intestine index (Figure 2D) of mice to varying degrees. In particular, the values for pre‐BFO‐M and pre‐BFO‐H groups were significantly higher (*p* < .05) than those observed for the CK group (approximately 30.18% and 37.46% as well as 17.89% and 22.36% higher, respectively). H&E staining (Figure 2E–H) of the colon tissue revealed the intact mucosal, submucosal, and muscular layers, regular arrangement of the crypts, deeper and longer crypts, and abundant goblet cells in the intestinal mucosa of the mice from the BFO groups as compared that of the mice from the CK group. These observations indicate that BFO does not exert any harmful effects on the intestinal function, instead mediate protective effects.

### 3.3 Effect of BFO treatment on colitis emergence and development in mice

**Figure 3:**
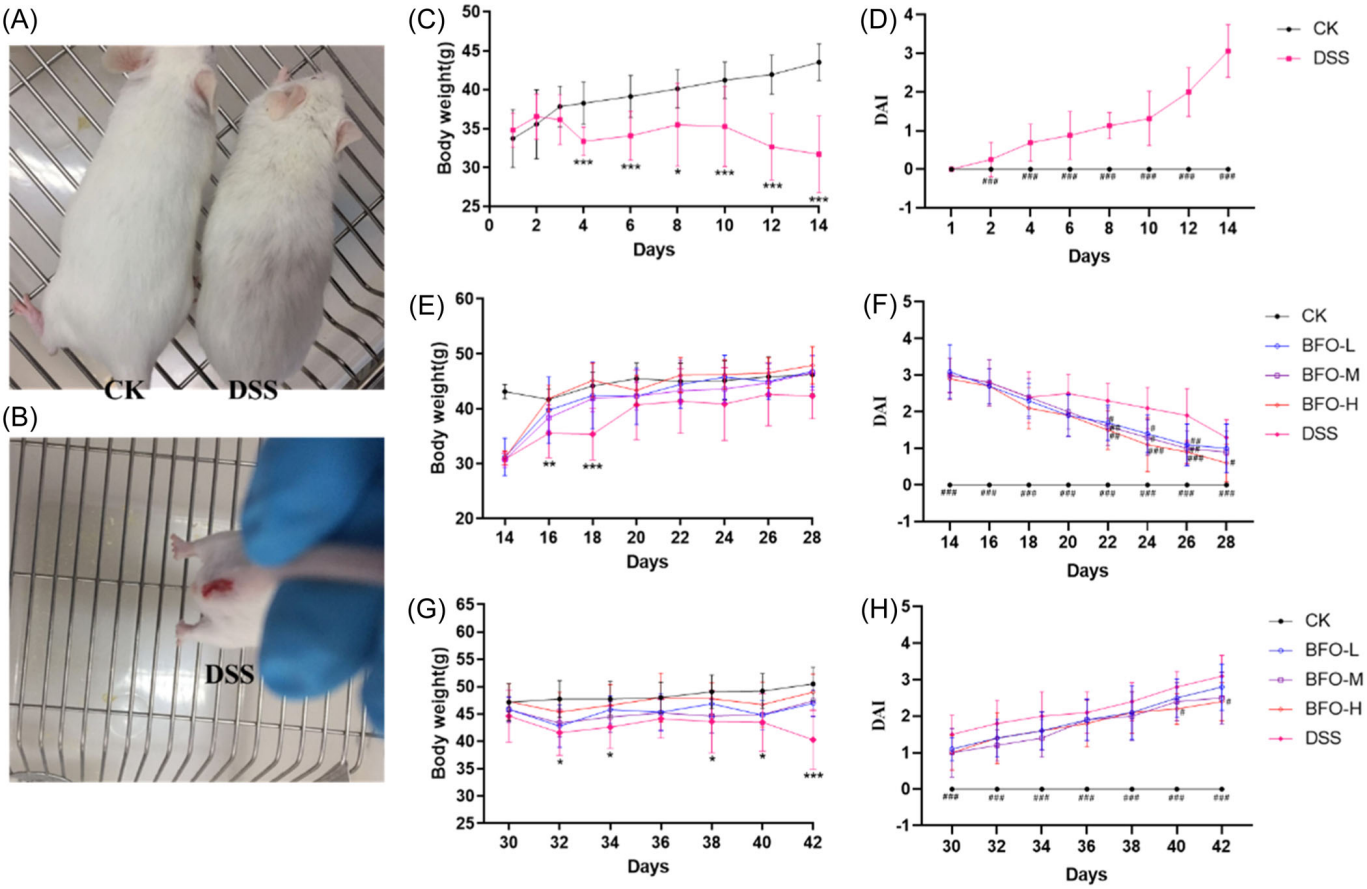
The appearance (A) and anal phenotype (B) of mice after the final administration of dextran sodium sulfate (DSS), and average body weight changes and disease activity index on Day 1–14 (C, D), 14–28 (E, F), and 28–42 (G, H). Data are means ± standard deviation (SD; *n* = 8). *p < .05, **p < .01, ***p < .001 versus CK group; ^#^ *p* < .05, ^##^ *p* < .01, ^###^ *p* < .001 versus DSS group (analysis of variance).

To evaluate the anti‐colitis effects of BFO, a mouse model of colitis was established using 3% DSS, and then orally treated with different concentrations of BFO. During the experimental period, changes in the body weight and DAI were daily recorded. In the model group, the mice showed pathological features such as piloerection, dim and matte hair, hogback (Figure 3A), visible bloody stools (Figure 3B), significant weight loss (Figure 3C), AND increased DAI (Figure 3D), indicating the successful establishment of a colitis model.

Weight change is considered as an indicator of severity of colitis in mice.^31^ In the second stage of the experiment, mice from all treatment groups began to regain their weights after the termination of DSS administration (Figure 3E). Interestingly, the body weight increased more rapidly for the mice fed with BFO than for the mice fed with water. In particular, during the first 4 days (Day 16 and 18) of DSS discontinuation, the body weights of the mice from the DSS group were significantly lower than those of the mice from the CK group. While there was no difference in the body weights between the BFO and CK groups, the effect was better in the BFO‐H group. DAI is an important parameter for evaluating the severity of colitis, including weight change, fecal occult blood, and stool occult blood.^32^ As expected, after Day 20, the mice from the BFO group showed a significant or extremely significant decrease in DAI scores as compared with those from the DSS group (Figure 3F).

In the third stage, we administered mice with DSS and different concentrations of BFO. The results showed that the body weights significantly decreased for the mice fed with DSS as compared to those from the CK group. On the other hand, the mice treated with different concentrations of BFO (BFO‐L, BFO‐M, and BFO‐H groups) could better maintain their body weights than those from the DSS group, especially the mice from the BFO‐H group (Figure 3G). Furthermore, BFO effectively alleviated the increase in the DAI. The BFO‐H group had significantly lower DAI than the DSS group, especially on Day 40 and 42 (approximately 21.43% and 22.58% lower, respectively) (Figure 3H). These results indicate that BFO can facilitate quick recovery from the symptoms caused by DSS and effectively reduce the severity of DSS‐induced colitis.

### 3.4 Inhibitory effects of BFO treatment on colon injury

**Figure 4:**
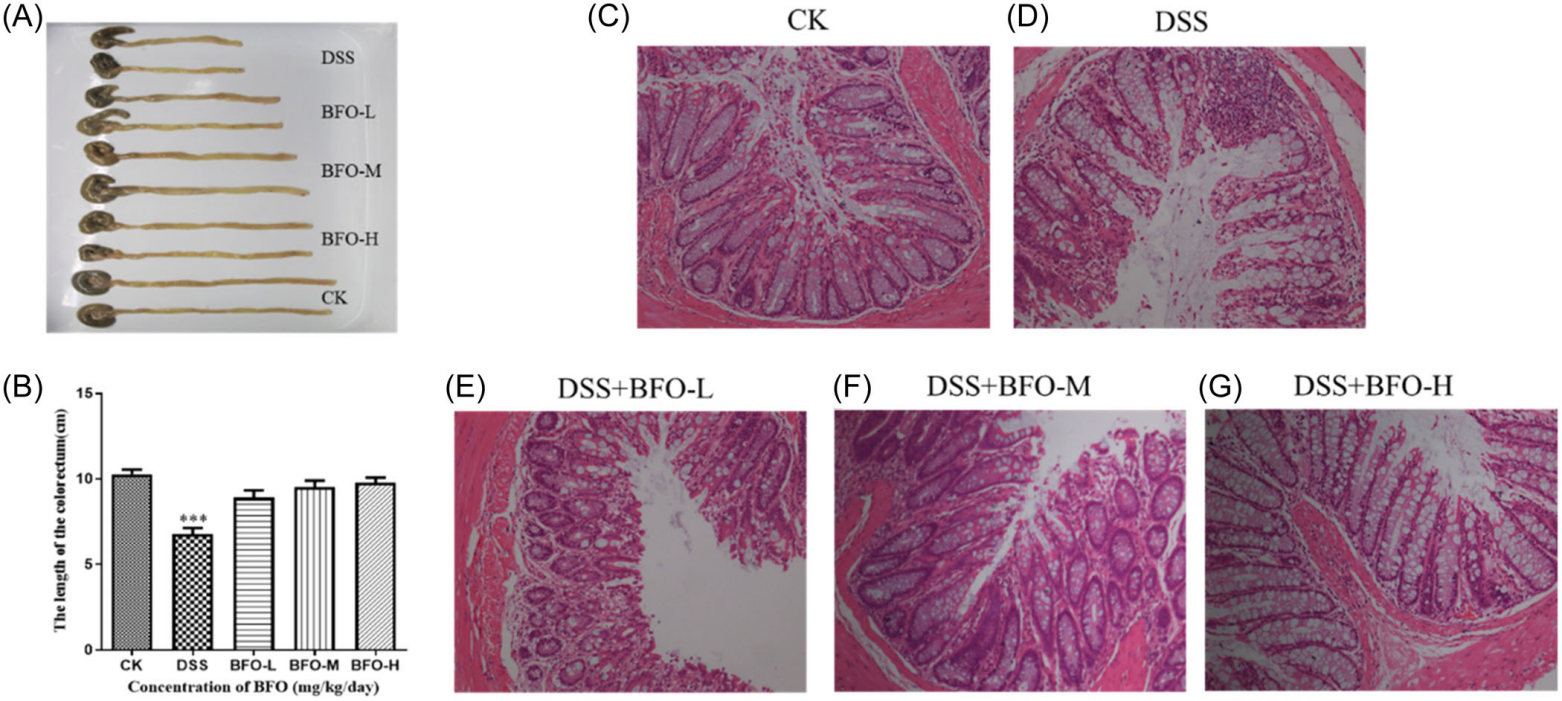
The effect of Burdock fructooligosaccharide (BFO) on the colon in the dextran sodium sulfate (DSS)‐induced colitis mice. (A) Macroscopic colon images, (B) colon lengths, and (C–G) H&E staining of colon tissues. Data are means ± SD (*n* = 5). ***p < .001 versus control group (CK) (analysis of variance).

In our study, the colon length was approximately 11.08 cm for normal mice at the end of the experiment. As expected, the colon lengths of the mice from the DSS group were approximately 38.81% lower than those of the mice from the CK group (Figure 4A,B). The colon lengths of the mice treated with different concentrations of BFO (BFO‐L, BFO‐M, and BFO‐H groups) were also lower than those of the mice from the CK group; the effect was dose‐dependent, but the difference was not significant (approximately 15.89%, 9.39%, and 6.32% lower, respectively). Therefore, BFO treatment could restore the DSS‐induced colon shortening and improve the symptom of colitis, with BFO‐H being the most effective treatment.

H&E staining results revealed the normal colonic morphology in the mice from the CK group (Figure 4C). DSS treatment disrupted the epithelial structure of the crypt, disturbed the organization of the mucosal layer, and damaged the integrity of goblet cells (Figure 4D). However, supplementation with various concentrations of BFO resulted in the amelioration of crypt distortion and intima irregularity, an increase in the number of goblet cells, and restoration of the structural organization induced by DSS to a level comparable to or surpassing that observed in the CK group (Figure 4E–G). These indicated that BFO can prevent the disruption of the intestinal barrier structure and protect the intestinal barrier function.

### 3.5 Effect of BFO treatment on the inflammatory responses of the colitis mice

**Figure 5:**
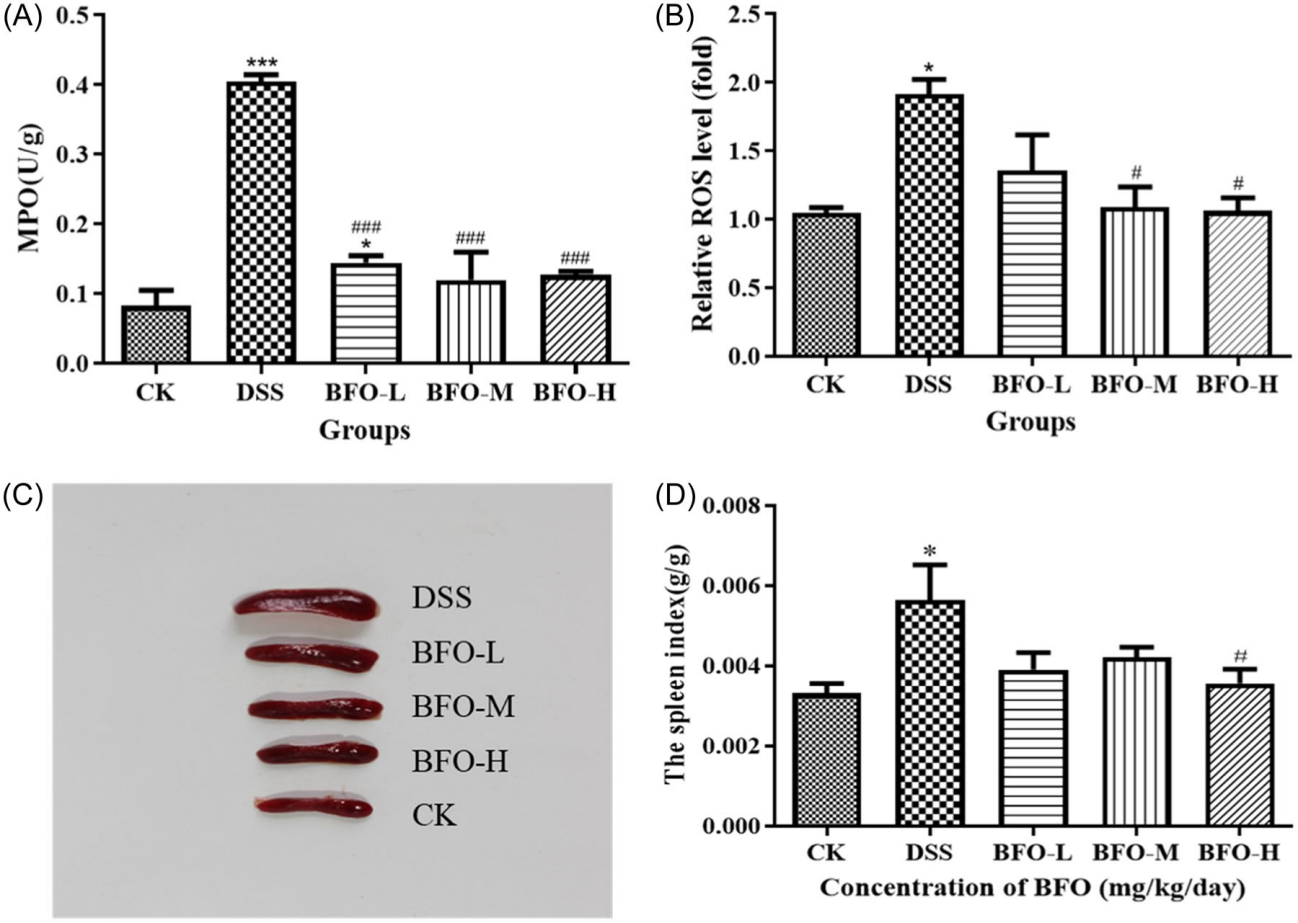
Effect of Burdock fructooligosaccharide (BFO) on colonic injury and inflammatory responses in the spleen. The changes of myeloperoxidase (MPO) activity (A) and the accumulation of reactive oxide species (ROS) (B). The macroscopic images of the spleen (C) and the spleen index scores (D). Experiments were repeated at least five times, data are presented as the mean ± SD. *p < .05, ***p < .001 versus CK group; ^#^ *p* < .05, ^###^ *p* < .001 versus dextran sodium sulfate (DSS) group (analysis of variance).

At the end of the experiment, we found that the MPO activity in colon tissues was extremely significantly higher in DSS groups than that in the CK group (Figure 5A). However, BFO administration significantly inhibited the increase in MPO activity induced by DSS (*p* < .001). Although the inhibitory effect in the BFO‐L group was slightly lower than that in the other BFO groups, it was higher than that observed in the CK group. There was no significant difference between BFO‐M and BFO‐H group as compared to the CK group. The values of BFO‐L, BFO‐M, and BFO‐H groups were approximately 74.57%, 44.65%, and 53.80% higher than that reported for the CK group, respectively.

As shown in Figure 5B, the production of ROS in colon tissues significantly increased following DSS stimulation and this effect was drastically reduced after BFO administration. The effects were more pronounced in BFO‐M and BFO‐H groups than in the other groups (Figure 5B). Thus, BFO effectively decreased the infiltration of neutrophils and the accumulation of ROS in the colon stimulated by DSS.

The administration of DSS resulted in an observed enlargement of the mouse spleen, whereas treatment with BFO effectively mitigated this effect (Figure 5C). We calculated the spleen index, and found that the spleen to body weight ratio was approximately 70.19% higher for the mice from the DSS group than for those from the CK group (*p* < .05) (Figure 5D). However, BFO treatment prevented the DSS‐induced increase in the spleen index. The values observed in BFO‐L, BFO‐M, and BFO‐H groups were approximately 30.82%, 25.28%, and 36.81% lower than the value reported for the DSS group, respectively. These results suggest that BFO can attenuate the immune organ abnormalities stimulated by DSS.

### 3.6 Effect of BFO treatment on the inflammatory cytokines of the colitis mice

**Figure 6:**
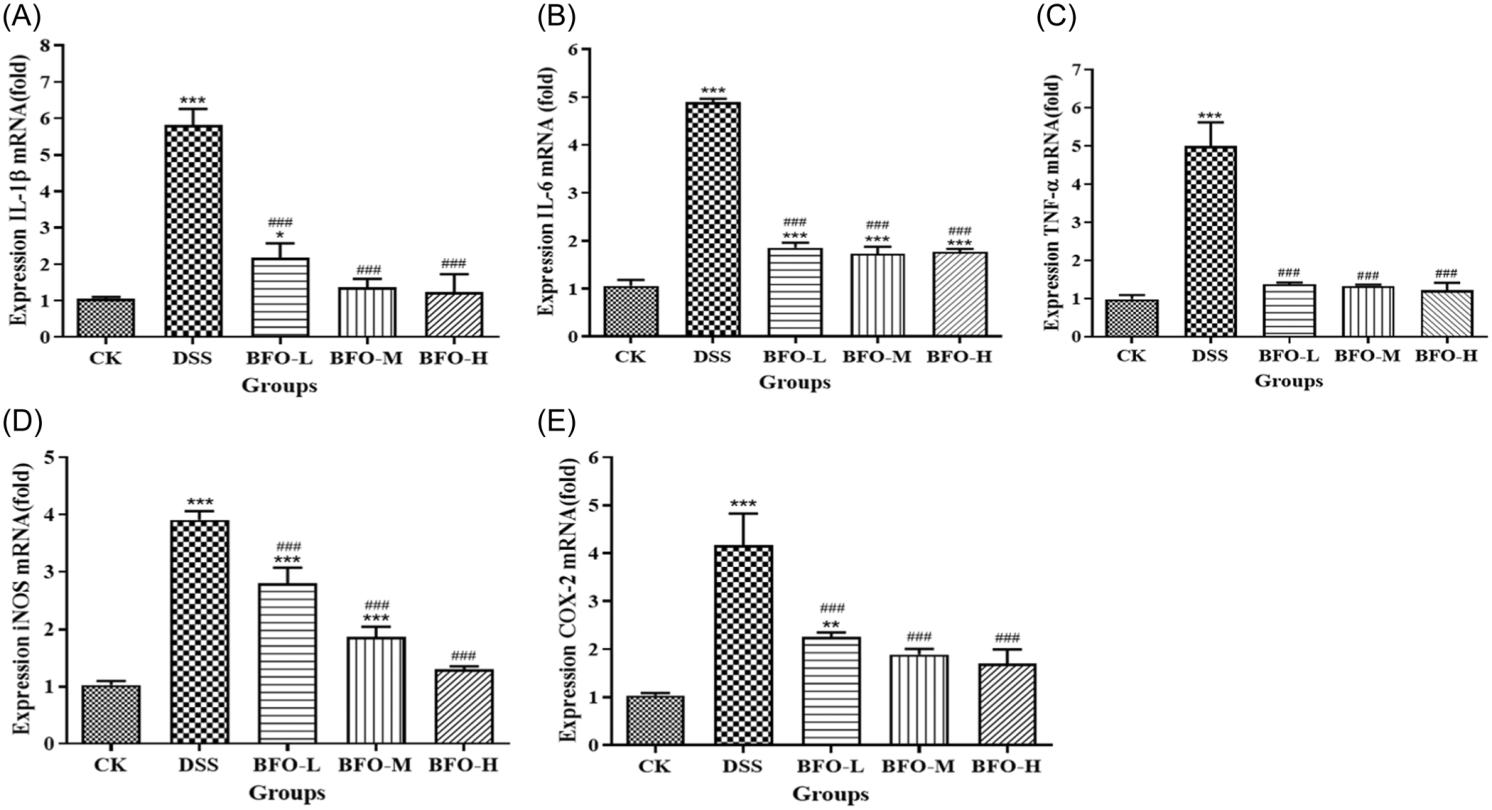
Effect of Burdock fructooligosaccharide (BFO) on the mRNA expression levels of inflammatory cytokines interleukin (IL)‐1β (A), IL‐6 (B), tumor necrosis factor‐α (C), inducible nitric oxide synthase (D), and cyclooxygenase 2 (E) in colon of the dextran sodium sulfate (DSS)‐induced colitis mice. Experiments were repeated at least five times, data are presented as the mean ± SD. *p < .05, **p < .01, ***p < .001 versus CK group;  ^###^ *p* < .001 versus DSS group (analysis of variance).

To investigate the effect of BFO on the inflammatory responses of DSS‐induced colitis mice, we determined the mRNA expression levels colon‐related genes, including *IL‐1β, IL‐6, TNF‐α*, inducible nitric oxide synthase (*iNOS*), and cyclooxygenase 2 (*COX‐2*), by qPCR (Figure 6). The expression of these inflammatory cytokines was significantly upregulated in DSS‐treated mice compared with that in the mice from the CK group (*p* < .01). Interestingly, BFO treatment significantly downregulated the expression of these inflammatory cytokines (TNF‐α, IL‐1β, and IL‐6) induced by DSS (*p* < .01); the best effect was recorded in the mice from the BFO‐H group. The administration of BFO significantly reduced the upregulation in iNOS expression induced by DSS in a concentration‐dependent manner (Figure 6D). Although there was no significant difference in COX‐2 expression among BFO‐L, BFO‐M, and BFO‐H groups (Figure 6E), different concentrations of BFO significantly reduced the upregulation in DSS‐induced COX‐2 expression.

**Figure 7:**
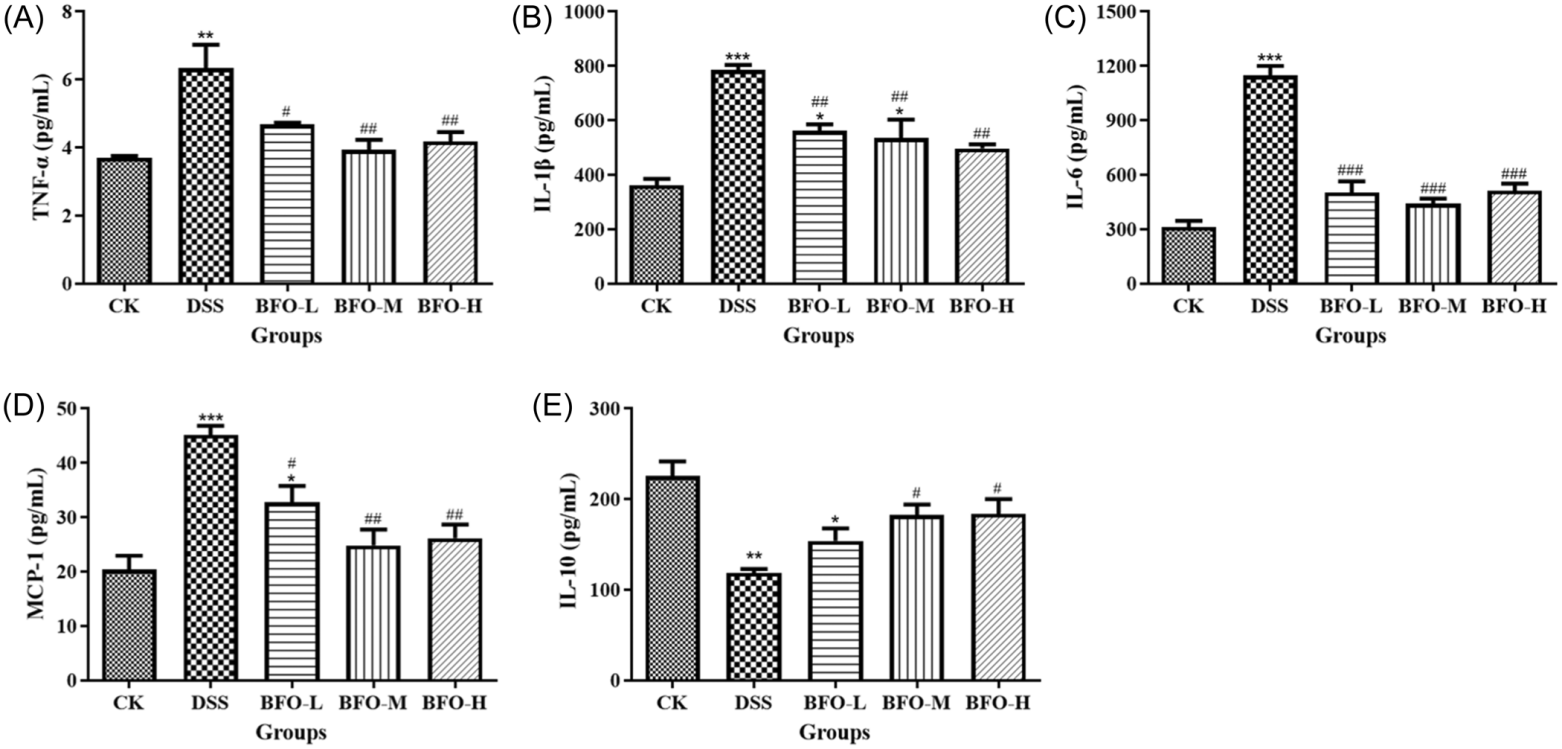
Effect of Burdock fructooligosaccharide (BFO) on the inflammatory cytokines tumor necrosis factor‐α (A), interleukin (IL)‐1β (B), IL‐6 (C), monocyte chemoattractant protein‐1 (D), and IL‐10 (E) in serum of the dextran sodium sulfate (DSS)‐induced colitis mice. Experiments were repeated at least five times, data are presented as the mean ± SD. *p < .05, **p < .01, ***p < .001 versus CK group; ^#^ *p* < .05, ^##^ *p* < .01, ^###^ *p* < .001 versus DSS group (analysis of variance).

Moreover, we observed a significant upregulation of serum Pro‐inflammatory cytokine levels following DSS administration, whereas treatment with BFO significantly attenuated the expression of inflammatory cytokines (TNF‐α, IL‐1β, and IL‐6) (Figure 7A–C), as well as monocyte chemoattractant protein‐1 (MCP‐1) (Figure 7D) TO varying degrees. The MCP‐1 levels in BFO‐L, BFO‐M, and BFO‐H groups were approximately 25.04%, 32.55%, and 35.97% lower than that in the DSS group (*p* < .05), respectively. The level of anti‐inflammatory cytokine IL‐10 in the DSS group was 47.39% lower than that in the CK group (*p* < .01). BFO treatment significantly restored the IL‐10 level, with an approximate increase of 29.91%, 53.80%, and 54.91% in the BFO‐L, BFO‐M, and BFO‐H groups respectively compared to the DSS group (Figure 7E).

## 4 DISCUSSION

Numerous studies have reported that the occurrence and development of UC are closely related to the intestinal barrier function and inflammatory response.^2^ Polysaccharides can alleviate UC by regulating the levels of both immune cells and inflammatory cytokines, reducing intestinal oxidative stress, protecting the intestinal mucosal barrier, and regulating the gut microbiota.^33^, ^34^ The BFO used in this study was an inulin‐type fructan with a purity of 99.439%, and was used to explore its protective effects on the colon after stimulation with DSS. Administration of BFO alone showed that it caused no damage to colons of Kunming mice, but instead increased the colon index, enhanced the index of the whole intestine, and prevented disruption of internal functional structures, especially in the Pre‐BFO‐H group (500 mg/kg/day).

The DSS‐induced colitis is frequently employed to investigate the pathogenesis of UC, encompassing manifestations such as weight loss, chronic diarrhea, bloody stool, and colon‐shortening.^35^, ^36^ Based on the results of BFO administration alone, a mouse model of colitis was established using 3% DSS in three stages. The first stage involved the establishment of the colitis model, in which the mice exhibited symptoms of piloerection and matte hair, bloody stools, chronic diarrhea, significant weight loss, and elevated DAI, consistent with the results reported by Song et al.^30^ and Shin et al.,^37^ indicating that the colitis model was successfully established. In the second stage, DSS administration was terminated, and the mice received oral doses of different BFO concentrations, upon which the symptoms of the mice in all treatment groups began to improve. Notably, the body weight increased more rapidly and DAI scores decreased more significantly in the mice fed with BFO than in those fed with water. These results indicated that BFO promoted rapid recovery from the effects of DSS. In the third stage, the mice were re‐exposed to DSS and concomitantly treated with different concentrations of BFO. The BFO‐treated mice were observed to be better able to maintain their body weights with mitigation of the elevated DAI compared with the DSS‐only‐treated group; these effects were especially evident in mice in the BFO‐H group (500 mg/kg/day). These results indicate that BFO can effectively protect mice from weight loss and reduce the severity of disease caused by DSS stimulation.

The shortening of colon length is another significant symptom of colitis, which exhibits a positive correlation with the severity of the disease. Moreover, restoration of colon length can be observed upon alleviation of inflammation.^38^, ^39^ At the end of the experiment, the colon lengths of the mice treated with different concentrations of BFO showed significant mitigation of the colon shortening induced by DSS, with the colons even restored to lengths similar to those of in the CK group. The effects were more pronounced in the BFO‐H group than in the other groups. Moreover, the disruption of intestinal barrier integrity as the main pathological feature of colitis.^40^ The dysfunction in the barrier function of the colon after DSS treatment of mice would increase colonic permeability, leading to the exposure of the epithelial cells to intestinal bacteria and their decomposition products and consequently exacerbating the progression of colitis.^41^ However, H&E staining showed that BFO led to the attenuation of the crypt distortion and intima irregularity, increment in the number of goblet cells, and restoration of the structural organization induced by DSS to a level comparable to or surpassing that observed in the CK group. Goblet cells in the intestine secrete mucus, which covers the surface of epithelial cells and plays a defensive role.^42^, ^43^ Thus, BFO exhibited the ability to prevent the disruption of the intestinal barrier structure caused by DSS and protect the intestinal defense function. This observation is in line with previous findings that demonstrated the ability of different kinds of polysaccharides to recover the intestinal barrier function.^44^

Neutrophils infiltrating the inflammatory tissue secrete MPO, an indicator of tissue injury and inflammation, which is excessively produced under DSS stimulation.^45^, ^46^ As shown in the results, the MPO activity was extremely significantly higher in DSS groups than in the CK group, consistent with the observation reported by Qiu et al.^45^ However, two cycles of BFO treatment resulted in an extremely significant reversal of the increased MPO activity induced by DSS (*p* < .001), with values close to those of the CK group. Furthermore, a similar trend was observed in the colon ROS. Wang et al.^47^ reported that the destruction of the colonic mucosal barrier structure and accumulation of neutrophils may lead to the overproduction of ROS, which is usually associated with the exacerbation of inflammatory disease. As expected, the production of ROS significantly increased following DSS stimulation. However, BFO effectively decreased the infiltration of neutrophils and the accumulation of ROS in the colon stimulated by DSS, thereby providing a better protection to the mucosal structure of the colon, with the BFO‐M and BFO‐H groups exhibiting greater efficacy as treatments.

The spleen is the most important lymphatic organ in the body, which can enlarge in response to infection or inflammation.^48^, ^49^ DSS increases the splenic weight and stimulate differentiation and proliferation of various immune cells,^50^, ^51^ consistent with the present observations. The spleen enlargements and increases in the spleen index observed in the DSS group were markedly reduced following administration of BFO. This observation may be attributed to the BFO‐mediated inhibition of colonic inflammation and the consequent protection of the immune organ. Together, these results suggest that BFO attenuated the DSS‐induced colonic tissue damage and immune organ abnormalities.

Pro‐inflammatory cytokines play a pivotal role in the progression of DSS‐induced colitis.^52^ In our results, DSS administration significantly upregulated the mRNA expression of pro‐inflammatory cytokines (IL‐1β, IL‐6, and TNF‐α) in the mouse colon. Interestingly, this upregulation was dramatically reduced after BFO administration. These observations are similar to the effects of *Scutellaria baicalensis* Georgi polysaccharide ^53^ and oyster polysaccharides reported in previous studies.^4^ iNOS and COX‐2 are involved in signaling pathways related to the inflammatory response, and their activation can induce the production of nitric oxide (NO) and prostaglandin E2 (PGE2) in the mucosa, respectively. This phenomenon may lead to the elevation in the production of inflammatory cytokines (TNF‐α, IL‐1β, and IL‐6), exacerbation of inflammation, and subsequent tissue damage.^54^, ^55^ The administration of BFO significantly reduced the upregulation in iNOS and COX‐2 expression induced by DSS in mouse colonic tissue in a concentration‐dependent manner, which might be related to the upregulation of inflammatory cytokines (IL‐1β, IL‐6, and TNF‐α), and also indicated that BFO can alleviate subsequent damage to the colon.

Xu et al.^8^ found that a sodium alginate‐based hydrogel loaded with lutein ameliorated the colon tissue damage in colitis mice by reducing levels of serum pro‐inflammatory cytokines (TNF‐α, IL‐1β, and IL‐6) and inhibiting nuclear factor kappa B (NF‐κB). Consistent with this study, DSS administration significantly increased the levels of serum pro‐inflammatory cytokines and that BFO treatment significantly reduced inflammatory cytokines (TNF‐α, IL‐1β, and IL‐6) to varying degrees. Similar effects were observed in our previous study ^28^ where BFO exerted anti‐inflammatory activities in a lipopolysaccharide (LPS)‐induced RAW264.7 macrophage model. Inflammatory cells such as neutrophils and macrophages not only eliminate foreign bodies and bacteria in the colon but also secrete inflammatory cytokines. Excessive and continuous secretion of inflammatory cytokines can cause colon tissue damage.^56^ MCP‐1, a key chemokine that regulates the migration and infiltration of monocytes,^57^ was also significantly upregulated after DSS stimulation. However, this increase was significantly reversed after BFO administration, which indicates that BFO can weaken the adhesion and aggregation of monocytes and macrophages in the mucous layer,^58^, ^59^ and consequently reduce the damage to the colonic mucosa. Thus, BFO attenuated the overexpression and excessive secretion of inflammatory cytokines induced by DSS, and consequently reduced intestinal inflammation. IL‐10 is an important anti‐inflammatory cytokine secreted by regulatory T‐cells (Tregs) and participate in maintaining intestinal homeostasis, its deficiency in mice can cause spontaneous colitis.^60^, ^61^ In our study, long‐term administration of DSS resulted in an extremely significant decrease in IL‐10 secretion, while BFO treatment was effective in maintaining IL‐10 levels, thereby dampening intestinal inflammation.

## 5 CONCLUSION

In conclusion, administration of BFO alone did not disrupt intestinal function; instead, it exerted a protective effect. In the experimental model of colitis, BFO was found to effectively prevent colon shortening and preserve the function of the intestinal barrier, thus effectively reducing the severity of DSS‐induced colitis by maintaining barrier integrity, inhibiting increased MPO activity, attenuating splenic abnormalities, and reducing the expression and secretion of pro‐inflammatory cytokines. Furthermore, BFO also exhibited superior ability to maintain IL‐10 levels, thus dampening intestinal inflammation. These findings enhance our understanding of the potential benefits of BFO in alleviating UC and provide a theoretical foundation for utilizing BFO in drug development or functional food products aimed at preventing colitis.

## AUTHOR CONTRIBUTIONS

Qunfei Ma and Xiujuan Zhang performed the research. Kaoshan Chen, Chunyan Liu, and Qiang Chen designed the research study. Yan Lu and Yiru Chen contributed essential reagents or tools. Qunfei Ma, Xiujuan Zhang, and Xuan Xu analyzed the data. Qunfei Ma and Xiujuan Zhang wrote the paper. Chunyan Liu and Kaoshan Chen revised the manuscript. All authors have thoroughly read and approved the final manuscript.

## CONFLICT OF INTEREST STATEMENT

The authors declare no conflict of interest.

## ETHICS STATEMENT

All experiments were performed in accordance with the principles of the National Guidelines for the Nursing and Use of Laboratory Animals and were approved by the Ethics Committee of Shandong University (No. SYDWLL‐2018‐12).

## Data Availability

The data that support the findings of this study are available from the corresponding author upon reasonable request.
